# Shared brain and genetic architectures between mental health and physical activity

**DOI:** 10.1038/s41398-022-02172-w

**Published:** 2022-10-03

**Authors:** Wei Zhang, Sarah E. Paul, Anderson Winkler, Ryan Bogdan, Janine D. Bijsterbosch

**Affiliations:** 1 Radiology Department, Washington University School of Medicine, St. Louis, MO USA; 2 Department of Psychological & Brain Sciences, Washington University in St. Louis, St. Louis, MO USA; 3 National Institute of Mental Health/National Institutes of Health, Rockville, MD USA

**Keywords:** Neuroscience, Genetics

## Abstract

Physical activity is correlated with, and effectively treats various forms of psychopathology. However, whether biological correlates of physical activity and psychopathology are shared remains unclear. Here, we examined the extent to which the neural and genetic architecture of physical activity and mental health are shared. Using data from the UK Biobank (*N* = 6389), we applied canonical correlation analysis to estimate associations between the amplitude and connectivity strength of subnetworks of three major neurocognitive networks (default mode, DMN; salience, SN; central executive networks, CEN) with accelerometer-derived measures of physical activity and self-reported mental health measures (primarily of depression, anxiety disorders, neuroticism, subjective well-being, and risk-taking behaviors). We estimated the genetic correlation between mental health and physical activity measures, as well as putative causal relationships by applying linkage disequilibrium score regression, genomic structural equational modeling, and latent causal variable analysis to genome-wide association summary statistics (GWAS *N* = 91,105–500,199). Physical activity and mental health were associated with connectivity strength and amplitude of the DMN, SN, and CEN (*r*’s ≥ 0.12, *p*’s < 0.048). These neural correlates exhibited highly similar loading patterns across mental health and physical activity models even when accounting for their shared variance. This suggests a largely shared brain network architecture between mental health and physical activity. Mental health and physical activity (including sleep) were also genetically correlated (|rg| = 0.085–0.121), but we found no evidence for causal relationships between them. Collectively, our findings provide empirical evidence that mental health and physical activity have shared brain and genetic architectures and suggest potential candidate subnetworks for future studies on brain mechanisms underlying beneficial effects of physical activity on mental health.

## Introduction

Mental health and physical activity have both been linked to emotion, cognition, and brain correlates [1–4]. Furthermore, physical activity is known to improve various psychiatric conditions [5–7]. Yet, it remains elusive whether mental health and physical activity overlap in neural and genetic architectures. Here, in a large population sample (*n* = 6389 from the UK Biobank [8]), we estimate the extent to which physical activity and general mental health share patterns of resting-state functional MRI network measures and genetic architectures.

### Potential overlap between mental health and physical activity in neural networks

Functional alterations in large-scale brain networks have been consistently implicated in a wide range of psychiatric disorders [9]. Dysfunctional configuration of neurocognitive networks such as the default mode, salience, and central executive networks (DMN, SN, CEN) has been hypothesized to characterize major psychiatric disorders including depression and anxiety [10]. In line with this hypothesis, findings from meta-analyses have shown that core cognitive and affective abnormalities in major depression can be accounted for by hypo-connectivity within the CEN and hyper-connectivity within the DMN [11], together with hypo-connectivity between the control systems (i.e., CEN) and salience, emotion processing systems (i.e., SN) [12]. Similarly, a recent meta-analysis suggests that anxiety disorders are characterized by hypo-connectivity between subcortical limbic circuits that partially overlap with the SN, CEN, and DMN, as well as decoupling between the CEN and DMN [13]. Furthermore, the personality trait neuroticism, which is considered a risk marker for psychopathology, has also been linked to alterations in functional brain networks [14]. These same networks have also been linked to physical activity. Evidence from fMRI studies on physical activity demonstrated the changes in the activity of and functional connectivity between these network hub regions including the hippocampus, parahippocampus, dorsal anterior cingulate cortex (dACC), and ventromedial prefrontal cortex (vmPFC) that primarily subserve executive functions such as working memory, attention, and inhibition [15–19]. At the more system level, a 12-month aerobic walking intervention was found to increase resting-state functional connectivity between subnetworks of DMN, and between subnetworks of SN [20], whereas connectivity in the CEN was found to increase after multiple sessions of high-intensity interval training [21]. Interestingly, the intensity of physical exercise appeared to modulate functional connectivity changes in the hub regions of the CEN [22], as well as in the DMN subsystems acutely and after 3 months of training [23].

Additionally, although sleep might not be considered a type of physical activity for its inactive nature, it is closely connected to mental health such that sleep problems have been considered a risk factor for subsequent development of depressive symptoms [24, 25], and that disrupted sleep is often seen in patients with various types of mood and anxiety disorders [26–28]. In relation to physical activity, sleep disturbance has been shown to reduce activity levels and increase the risk of exercise-related injuries [29]. Despite its close relationships with both mental health and physical activity, sleep has been largely overlooked in the literature for physical activity in relation to mental health. Currently, sleep duration can be accurately measured with ecological sampling approaches such as wrist-worn accelerometers, which have been adopted in medical and mental health research [30, 31]. At the neural network level, sleep deprivation has been linked to a reduction in functional connectivity within the DMN [32–34], whereas increased sleep duration has been linked to strengthened connectivity within the DMN but reduced connectivity between DMN and SN [35]. Fluctuations in arousal (i.e., an indication of drowsiness) during resting-state scan acquisitions have also been linked to the fMRI signal amplitude in sensorimotor networks [36]. These studies together highlight the engagement of the DMN, SN, and CEN in both mental health and physical activity and demonstrate that the interplays between these large-scale intrinsic networks and subnetworks are coupled with changes in mental health symptoms and physical exercises. Yet, it remains unclear whether similar connectivity patterns or signal changes of these networks are shared by mental health and physical activity.

### Potential overlap between mental health and physical activity in genetic variance

Physical activity is known to promote resilience to various psychiatric conditions, alleviating symptoms of depression, anxiety, and negative mood [37, 38]. This buffering effect may be rooted in the shared genetic variance between mental health and physical activity. For instance, recent research employing summary statistics from independent genome-wide associate studies (GWAS) showed that higher polygenic risk scores for depression are associated with increased odds of incident depression, whereas self-reported physical activities such as walking, jogging, running, dancing and yoga appeared to reduce the odds with similar magnitude [39]. This buffering effect has also been observed using more objective measurement of physical activity such that reduced activity levels measured by accelerometer were found to associate with diagnoses of schizophrenia, bipolar disorder, depression, or autism spectrum disorders (ASD) and that healthy participants without disorder diagnoses were observed to perform the less physical activity if they had a higher polygenic risk score for schizophrenia, depression, and ASD [40].

Additionally, overlapping genetic architectures may exist across various psychiatric disorders including anxiety and depression [41–43] as psychiatric phenotypes are highly polygenic [44]. Thus, latent genetic factors capturing shared variance across clusters of psychiatric symptoms is hypothesized to improve identification of associations between mental health and physical activity. For instance, genomic contributions to disorders such as depression and anxiety are captured by a genetic factor for internalizing disorders that are primarily characterized by these two disorders as indicated by confirmatory factor analysis, and this genetic factor is positively genetically associated with various adverse health outcomes and negatively genetically associated with physical movement patterns [45]. These findings point to the possibility that the genetic architectures of mental health and physical activity may overlap and the degree and phenotypic specificity of overlap remain to be tested.

### Shared brain and genetic architectures?

Together, the studies reviewed above show that mental health and physical activity both involve large-scale brain networks such as the DMN, SN, and CEN. Additionally, mental health and physical activity may have partially overlapping genetic architectures, with evidence showing associations between genetic liabilities of psychiatric disorders and physical activity, as well as genetic associations between latent factors of psychopathology and physical activity. As mental health and physical activity are also tightly related at the behavioral level (e.g., emotion, cognition), it is reasonable to speculate that these two constructs may partially overlap in the underlying neurobiological mechanisms. In this study, we aim to determine whether mental health and physical activity have shared variance in the brain and genetic architectures, using brain network measures and genomic summary statistics.

## Methods and materials

### Participants

The UK Biobank (UKB) is an openly accessible population dataset with neuroimaging data collection, in addition to extensive demographic, behavioral, lifestyle, and cognitive measures [8, 46]. An initial sample of *N* = 8378 participants from the UK Biobank was considered for this study. These participants had participated in accelerometer-based physical activity evaluations and visited the assessment center, where the resting-state fMRI and mental health questionnaire data were acquired. Data quality assurance resulted in exclusion of *N* = 64 participants for insufficient accelerometer data (see details below in section 2.2.2), and *N* = 1925 participants with considerable missing data in the mental health questionnaire (see details below in section 2.2.1). The final sample had *N* = 6389 participants with 2994 (46.9%) females (sample mean age = 63.74 ± SD 7.53). All participants provided informed consent. UK Biobank has ethical approval from the North West Multi-Center Research Ethics Committee (MREC). Data access was obtained under UK Biobank application ID 47267.

### Data acquisition and preprocessing

#### General mental health measures

The UKB general mental health questionnaire consisting of 41 items was conducted on the same day as fMRI data acquisition (https://biobank.ndph.ox.ac.uk/showcase/label.cgi?id=100060). This self-reported questionnaire primarily measured depression, anxiety, and neuroticism, as well as subjective well-being. These variables had varying degrees of missing data points partly due to responses such as “do not know”, “prefer not to answer”, or “none of above,” and partly due to question dependencies. To ensure robust model estimation while maximizing statistical power, individual variables or questions that had >30% missing values were excluded (*N* = 10; see full descriptions for each included an individual question in Table S1). A multivariate imputation procedure was then leveraged to handle the missingness in the remaining data. By default, this procedure implements multiple imputations with separate imputation models for each incomplete variable [47]. The predictive mean matching (PMM) approach was employed for imputing continuous variables, which first estimates a linear regression model for the target variable (e.g., Y) from all other variables in the data (e.g., non-Y variables) with complete observations. New coefficients are then drawn from the posterior predictive distribution of the estimated regression coefficients and used to calculate the predicted values for the missing entries in Y. The predictive values for the observed Y are also calculated using the estimated regression coefficients. Finally, a small set of candidate donors is formed from the observed Y (i.e., usually three or five donors) that have the closest predicted values to the missing Y, and the observed value from one donor will be randomly selected to replace the missing value [47]. Using PMM, 20 iterations were performed for each incomplete variable of mental health, and the final imputed value for any given missing entry was averaged across all iterations.

As the current mental health questionnaire covers a broad range of multiple constructs, including depression and anxiety symptoms, neuroticism, and subjective well-being, we performed data decomposition using principal component analysis (PCA) on the imputed data to extract the most relevant information about general psychopathology, using a R package (see details below in section 2.4). We retained the top principal components that collectively explained >50% variance of the data in the subsequent statistical analyses. PCA loadings of each individual question per component can be found in Table 1.

**Table 1:** Correlations between mental health and physical activity measures.

Physical activity	Mental health						
	PC1	PC2	PC3	PC4	PC5	PC6	PC7
**Overall**							
Sleep duration	−0.08^*^	0.04^*^	0.01	0.00	0.04^*^	−0.01	0.03
Sedentary	−0.03^*^	0.07^*^	0.07^*^	−0.02	−0.02	−0.03	0.03^*^
Light tasks	0.02	−0.02	0.01	−0.01	−0.04^*^	−0.01	0.00
Moderate	−0.02	−0.06^*^	0.02	0.01	0.03^*^	0.04^*^	0.01
Walking	0.01	−0.01	0.02	−0.03^*^	−0.06^*^	0.00	0.04^*^
Overall activity	−0.06^*^	0.04^*^	0.03^*^	−0.02	0.04^*^	0.01	0.08^*^
MET	−0.06^*^	0.10^*^	0.07^*^	−0.03	−0.01	−0.05^*^	0.02
**Weekdays**							
Sleep duration	−0.08^*^	0.04^*^	0.02	0.00	0.03^*^	−0.01	0.03^*^
Sedentary	−0.05^*^	0.08^*^	0.08^*^	−0.01	−0.04^*^	−0.02	0.04^*^
Light Tasks	0.01	−0.02	0.03	−0.03	−0.03^*^	−0.01	0.01
Moderate	−0.02	−0.05^*^	0.03	0.01	0.02	0.03^*^	0.03
Walking	0.02	0.00	0.04^*^	−0.04^*^	−0.09^*^	−0.01	0.04^*^
Overall activity	−0.05^*^	0.04^*^	0.06^*^	−0.04^*^	−0.01	0.00	0.07^*^
MET	−0.06^*^	0.11^*^	0.08^*^	−0.03	−0.07^*^	−0.04^*^	0.02
**Weekend**							
Sleep duration	−0.06^*^	0.04^*^	0.00	0.00	0.02	−0.01	0.02
Sedentary	−0.02	0.05^*^	0.06^*^	−0.02	−0.01	−0.02	0.02
Light tasks	0.02	−0.01	0.00	−0.01	−0.05^*^	−0.02	−0.02
Moderate	−0.02	−0.04^*^	0.01	0.00	0.01	0.04^*^	0.00
Walking	0.02	0.00	0.02	−0.03^*^	−0.06^*^	0.00	0.04^*^
Overall activity	−0.05^*^	0.04^*^	0.03	−0.02	0.03^*^	0.01	0.06^*^
MET	−0.05^*^	0.08^*^	0.05^*^	−0.02	0.01	−0.04^*^	0.02

Phenotypes of mental health were represented by the first seven principal components (i.e., PC1-PC7) that altogether explained 51.05% variance. Significant correlations are indicated by * after FDR corrections (i.e., all corrected *p*’s ≤ 0.045), and negative correlation coefficients are highlighted with light gray shading.

#### Physical activity measures

Accelerometer data were acquired for a subset of UKB participants during a seven-day monitoring period (https://biobank.ndph.ox.ac.uk/ukb/label.cgi?id=1008). This enabled real-time measuring of physical activity for the participants throughout the entire week. Following a recommendation for quality control [48], data from participants who had less than 72h device wearing time or had no data in each one-hour period of the 24h cycle were excluded (*N* = 64). Using a publicly available machine learning algorithm, we extracted measures of five types of physical activity including sleep, sedentary, walking, light task, and moderate activities [48]. This algorithm applied random forest and hidden Markov models to a 126-dimental vector that represented a range of time and frequency domain features for every non-overlapping 30-sec epoch. The resulting probability of each physical activity was then defined as the count of predicted activity type per 30-sec epoch divided by the number of epochs [48]. In addition to these probability measures, the average acceleration magnitude, and metabolic equivalents of task (MET) were included to indicate overall activity intensity. The mean values of these features were calculated across weekdays and weekends, respectively, as well as across the entire monitoring period (i.e., average over weekdays and weekends). To account for variation in each individual physical activity measure at different time points, all mean values were standardized by standard deviations for each participant. In total, 21 standardized physical activity measures were included in our analyses.

#### Resting-state fMRI data and brain network measure

Resting-state fMRI was acquired using a multiband sequence with an acceleration factor of 8 (TR = 0.735; voxel size = 2.4 × 2.4 × 2.4 mm3). Preprocessing steps included motion correction, grand-mean intensity normalization, high-pass temporal filtering, unwarping and ICA-FIX denoising (Alfaro-Almagro et al., 2018). Full details can be found in UK Biobank Brain Imaging Documentation (https://biobank.ctsu.ox.ac.uk/crystal/crystal/docs/brain_mri.pdf).

For this study, we used IDPs (imaging-derived phenotypes) that were generated and released by the UKB [46]. Specifically, partial connectivity matrices and network amplitudes from ICA with dimensionalities of 100 (ICA100) were considered (see Supplementary Results for the comparison with ICA25). ICA is a data-driven approach that can estimate the resting-state brain networks reliably and reproducibly, including the three networks of interest. It further helps eliminate noise in the data by separating noise and signal components [49]. These advantages of ICA make it an unbiased and powerful technique to study resting-state networks. We mapped the DMN, SN, and CEN onto the ICA components and then calculatied partial connectivity matrices and network amplitudes, as described below.

##### Mapping the networks of interest

We used the Stanford FIND atlas [50] to construct canonical spatial maps as the reference to identify the three brain networks of interest, namely the DMN, SN, and CEN in our sample. Notably, the FIND atlas is a functional connectivity-based parcellation atlas, from which we selected seven functional parcels that are all part of these three intrinsic networks, including dorsal (i.e., PCC and medial prefrontal cortex) and ventral default mode networks (i.e., retrosplenial cortex and medial temporal lobe), precuneus network, anterior (i.e., anterior insula and dorsal anterior cingulate cortex) and posterior salience networks (i.e., posterior insula), as well as left and right executive control networks (i.e., dorsolateral prefrontal cortex and parietal cortex in the left and right hemispheres respectively). These selected parcels well represent the subsystems of the three intrinsic networks that have been associated with physical exercises (e.g., via cognitive and interoceptive processing) [51, 52] and various psychiatric disorders including depression and anxiety [53–55]. Therefore, we selected them as our reference networks. To identify ICA components that can be mapped onto these networks of interest, we examined fifty-five signal components that were generated from ICA100 and detected the best matching components corresponding to each of seven selected network parcels, based on spatial correlations. Seven ICA components showing the highest spatial correlations with the FIND atlas were identified (mean *r* = 0.37). We refer to these ICA components as ‘subnetworks’ from the large-scale DMN, SN, and CEN as they represented the subsystems of the three intrinsic networks.

##### Extracting subnetwork edges

Dual regression was performed to obtain timeseries for each ICA component or subnetwork at the individual level [56]. These extracted subnetwork timeseries were then used to calculate the partial connectivity matrix and amplitude measures of interest. As described in Miller et al. [46], pairwise partial correlation coefficient were estimated using L2-regularized partial correlations between all 55 signal components from ICA100, which are deemed to be non-artifactual. This analysis resulted in a 55 × 55 partial correlation matrix, from which we selected the correlation coefficients corresponding to our seven components of interest (i.e., subnetwork edges). In total, 21 subnetwork edges were extracted and included in the subsequent analyses.

##### Calculating subnetwork amplitudes

In additional to pairwise partial correlations between these seven subnetworks, we further considered the signal amplitude of each subnetwork as the brain network variables. The amplitude is defined as the standard deviation of the ICA component timeseries [36]. Previous work has shown that amplitudes capture the overall signal fluctuations within each subnetwork and can offer complementary information in relation to behavioral measures. For example, a recent study using the UK Biobank dataset demonstrated independent associations between network amplitudes and behavioral measures in addition to connectivity strength [46]. Thus, we also included seven subnetwork amplitudes in the subsequent analyses.

##### Overview of resting-state measures

**Fig. 1:**
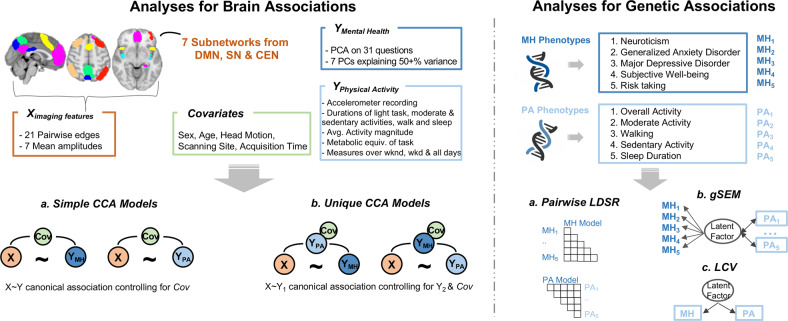
Overview of variables and analyses. Separate analyses were conducted for brain (left panel) and genetic associations (right panel). For brain associations, canonical correlation analysis (CCA) was employed for mental health (MH), and physical activity (PA) separately. Simple and unique CCA models only differed in whether the shared variance between MH and PA was accounted for. For genetic associations, GWAS summary statistics for five mental health phenotypes (MHP_1_-MHP_5_) and for five physical activity phenotypes (PAP_1_–PAP_5_) were leveraged into pairwise linkage disequilibrium score regression (LDSR) analyses, in MHP and PAP models separately. Genomic structural equation modeling (gSEM) was also employed to identify genetic associations between a latent factor from MH phenotypes and each PA phenotype, followed by a latent causal variable analysis (LCV) that allows for inferring causal genetic relationships among MH and PA phenotypes.

In summary, subnetwork edges from the partial connectivity matrix indicate the connection strength between each pair of seven subnetworks while controlling for all other ICA components, whereas subnetwork amplitudes capture the variance of signal changes within each subnetwork. In total, 28 brain measures including 21 partial connectivity strength measures (i.e., subnetwork edges) and seven amplitudes were included (see the overlayed subnetworks in Fig. 1; also see subnetwork selection in Fig. S1). All of these resting-state imaging measures are available from the UK Biobank showcase (bulk field IDs 25753 and 25755).

### Statistical analysis

Separate statistical analyses were performed to examine the shared brain network architecture and genetic architecture between mental health and physical activity (see Fig. 1 for an overview of the relevant variables and analyses).

#### Shared variance between mental health and physical activity

Pearson’s correlation was used to identify the shared variance between phenotypes of mental health and physical activity. As we decomposed the data of mental health into principal components, all correlations were performed using the individual-specific component scores. We further calculated the false discovery rate (FDR) to account for multiple testing on all pairwise correlations between mental health and physical activity phenotypes.

#### Brain associations with mental health and physical activity

Canonical correlation analysis (CCA) has been recognized as a key tool for population neuroimaging that allows for investigating associations between imaging and non-imaging variables [57]. Here in this study, CCA was performed to investigate the associations of brain measures with physical activity and with mental health separately (i.e., simple CCA models). Specifically, CCA finds a linear combination of brain measures that is maximally correlated with a linear combination of mental health or physical activity variables respectively, as defined in *Y* * *A* = *U* ~ *V* = *X* * *B* [58] where *Y* is the set of brain measures, *X* the set of mental health or physical activity measures, *A* and *B* are the linear weights, and *U* and *V* the canonical variables or canonical variate pair. The canonical correlation for each pair of canonical variates is defined as the correlation between *U* and *V*. Canonical loadings that indicate the shared variance between the original observations and canonical variables are calculated as the correlations between *Y* and *U*, or between *X* and *V*.

To further identify unique brain associations with physical activity and with mental health respectively, variance in brain network data explained by one set of variables was partialled out in the CCA model for the other set of variables (i.e., the unique CCA model included physical activity measures as covariates in the model for assessing brain-mental health associations and vice versa). Confounding variables (see details below) were included in all four CCA models and statistical inference for CCA results was made via 1000 permutations (i.e., breaking correspondence of participant identity with brain measures and mental health/physical activity measures), as implemented in the permCCA package [57]. Notably, the CCA model with the largest number of non-confounding variables included 28 brain measures and 21 physical activity measures, resulting in a ratio of ~130 observations (i.e., individuals) per feature. This is expected to ensure sufficient stability for our study [59].

To further investigate whether the patterns of brain measures in relation to mental health and physical activity overlap, posthoc analyses were carried out to test the significance of canonical loadings for each individual brain variable. Specifically, we aimed to determine whether the same brain measures contributed significantly to the canonical associations both with mental health and physical activity, and thus could indicate a shared brain basis. These analyses were conducted only for significant canonical variates within each individual CCA model. Significance of canonical loadings was inferred using permutation testing, where the correspondence between brain measures and mental health/physical activity measures for each individual participant is shuffled. Specifically, canonical loading for each brain variable was recorded per permutation, which resulted in separate null distributions of loadings for each brain variable. The loadings from the true (unpermuted) CCA were then compared against the matching null distributions for each individual brain variable. Statistical significance was determined as the proportion of permuted loadings equal to or higher than the observed loadings from the unpermuted analysis, divided by the total number of 1000 permutations. These permutation-derived p values were further corrected for the number of significant canonical variates within each model (i.e., record the permuted loadings across canonical variates). To compare brain variable patterns across different CCA models, we matched the first significant canonical variates from each model based on the correlations between the canonical variate for the brain measures (i.e., correlating the vector *U* obtained from the mental health CCA models with the vector *U* obtained from the physical activity CCA models).

To characterize the individual mental health questions and physical activity types in relation to the tested brain associations, we further examined the loading patterns of each individual question and physical activity type for the first canonical variate from all models, without testing for statistical significance.

#### Shared genetic architecture between mental health and physical activity

Genetic correlations between mental health and physical activity were examined by leveraging GWAS summary statistics for the relevant phenotypes.

##### Summary statistics

The mental health questionnaire used in this study includes items that measure neuroticism, anxiety, subjective well-being, depression, and risk-taking. We, therefore, sought to obtain summary statistics for these psychopathological phenotypes. First, we extracted summary statistics for *Neuroticism* from a GWAS meta-analysis of self-reported neuroticism in the UKB (using the same questions as in our study) and Psychiatric Genetics Consortium (using the NEO-FFI personality inventory) [60]. For *Generalized Anxiety Disorder*, we leveraged summary statistics from a GWAS of self-reported Generalized Anxiety Disorder 2-item scale scores in the Million Veteran Program [61]. We further obtained summary statistics for *Subjective Well-Being* from a GWAS meta-analysis of life satisfaction, positive affect, or both life satisfaction and positive affect across 59 cohorts [62]. For *Major Depressive Disorder*, we meta-analyzed summary statistics from case-control GWAS in the UK Biobank and Psychiatric Genomics Consortium [63] and the Million Veteran Program [64] (see Supplementary Results for further details). Lastly, we obtained summary statistics for *Risk Taking* from a GWAS study using the UKB data, which included the same question of risk-taking as in our study [65]. Although these psychopathological phenotypes obtained from the independent GWAS studies were not directly equivalent to the phenotypes derived from the mental health questionnaire in our brain association analyses due to different measurements, the underlying constructs of depression, anxiety disorders, neuroticism, subjective well-being, and risk-taking are identical. The phenotypes obtained to conduct genetic association analyses are therefore similar to those in the brain association analyses.

Summary statistics for accelerometer data-derived physical activity phenotypes, including moderate activity, overall activity, sedentary activity, walking, and sleep duration were derived from a GWAS of *N* = 91,105 participants of European ancestry in the UK Biobank [48]. The exact phenotypes were also included in our brain association analyses.

Please refer to Table 2 for an overview of all the summary statistics used in this study, including sample size and SNP heritability.

**Table 2:** Summary statistics from GWAS studies.

Reference	Phenotype	Dataset	Sample size	SNP *h*^2^
Nagel et al. 2018 [60]	Neuroticism	UK Biobank & PGC	390,279	0.10
Levey et al. 2020 [61]	GAD	Million Veteran Program	199,611	0.056
Okbay et al. 2016 [62]	SWB	Meta-analysis across 59 cohorts	298,420	0.040
Howard et al. 2019 [63]	MDD	UK Biobank & PGC	500,199	0.11
Levey et al. 2021 [64]		Million Veteran Program	250,215	
Linnér et al. 2019 [65]	Risk-taking	UK Biobank	431,126	0.050
Doherty et al. 2018 [48]	Physical activities	UK Biobank	91,105	0.10–0.21

*GAD* general anxiety disorder, *SWB* subjective well-being, *MDD* major depressive disorder, *PGC* Psychiatric Genetics Consortium.

##### Genetic correlations

We used linkage disequilibrium score regression (LDSR) and genomic structural equation modeling (gSEM) to test whether the genomic architecture associated with general mental health is shared with physical activity. LDSR leverages GWAS summary statistics to estimate genetic correlations by regressing the SNP statistics on the SNP linkage disequilibrium (LD) scores, or correlations between nearby genomic loci due to population stratification (i.e., systematic differences in allele frequencies due to differences in ancestry). gSEM characterizes the latent genetic architecture across phenotypes based on the LDSR-derived genetic correlation matrices [66]. To this end, we first applied LDSR to existing GWAS summary statistics of psychopathological phenotypes (i.e., neuroticism, generalized anxiety disorder, subjective well-being, major depressive disorder, and risk-taking) and physical activity phenotypes (i.e., overall activity, moderate activity, sedentary activity, sleep duration, and walking), respectively, to estimate pairwise genetic correlations within each construct (i.e., within mental health and within physical activity, respectively). We also examined genetic correlations between mental health phenotypes and physical activity phenotypes adjusted for sex and BMI. We then applied gSEM to the covariance matrix of psychopathology and that of physical activity separately, allowing one single latent factor to load freely within each model. Metrics indicating model fit (i.e., CFI, comparative fit index; SRMR, standardized root mean squared residual) and factor loadings from each of these models were used to determine whether one common genetic factor fit the physical activity and mental health data well, respectively.

Because our results indicated a poor model fit for some gSEM analyses (see details below in Results section 3.3), we focused on the model of mental health, where “risk-taking” was excluded to generate a latent factor of “negative affect” across other phenotypes. Specifically, we explored genetic correlations between the latent factor of “negative affect” (i.e., without risk-taking) and each of the five physical activity phenotypes. In addition, we examined genetic correlations between “risk-taking” alone and each individual physical activity phenotype, using LDSR. FDR correction was used to correct for multiple testing (*N* = 10 tests). We also repeated analyses that returned significant results, with adjustment for sex and BMI. Adjusted summary statistics from Doherty and colleagues (2018) were used in these analyses.

##### Causal relationships

To examine plausible causal associations between physical activity phenotypes and negative affect, we conducted a Latent Causal Variable Analysis (LCV). This approach finds a latent variable that mediates the genetic correlation between two traits, such as negative affect and sleep duration. Generally, if the latent variable has a stronger genetic correlation with trait 1 (e.g., sleep duration) than with trait 2 (e.g., negative affect), part of the genetic component of trait 1 is thought to be causal for trait 2. This partial causality can be quantified using the genetic causality proportion (GCP) of trait 1 (sleep duration) on trait 2 (negative affect), which can range between 0 (no partial genetic causality) and 1 (full genetic causality) [67]. The advantages of using LCV over the more traditional Mendelian Randomization for causal inference include increased power by leveraging SNPs across the genome and less susceptibility to confounding by horizontal pleiotropy [67].

### Confounding variables

Based on the literature, BMI, smoking, and drinking status were included as confounding factors in all statistical analyses for brain associations [68, 69]. Age, sex, head motion during rs-fMRI acquisition (i.e., mean frame-wise displacement), time difference in days between accelerometer recording (i.e., start date) and assessment center visit date (i.e., acquisition date for both mental health questionnaire and rs-fMRI), as well as the scanning site were further included. Due to varying degrees of missingness in the confound variables (i.e., up to 22%), the same imputation procedure, as described in *Section 2.2.2*, was performed except that additional usage of multinomial logistic regression was employed to impute categorical data with more than two levels (i.e., smoking and drinking status). Complete observations from all variables including the confounding variables were used in the imputation procedure. When calculating the final predicted values to replace missing data, the predicted values for continuous variables were averaged across 20 iterations and the level with the highest count across iterations was selected. All categorical variables after imputation were dummy coded for subsequent statistical analyses.

In the genetic correlation analyses, to ensure robust test effects, we repeated models with significant results including sex and BMI as covariates.

## Results

### Correlations between mental health and physical activity measures

In this sample, phenotypic measures of mental health and physical activity demonstrated overall small but significant correlations, with coefficients ranging between –0.08 and 0.11 (FDR-corrected *p* values ≤0.045). As shown in Table 1, the mental health measures (i.e., principal component scores of the seven principal components) were broadly associated with all physical activity measures. In particular, sleep showed the strongest association with the first principal component (PC1) of mental health such that greater sleep duration was associated with poorer overall mental health (i.e., loadings of PC1 were mostly negative as shown in Table S1), and walking had the greatest association with the fifth principal component (PC5) of mental health such that higher depression scores were linked to reduced walking time. The overall coefficient patterns were largely consistent for correlations between physical activity and mental health measures despite minor differences across different time windows (i.e., overall, weekdays, weekend; see full correlations in Table 1).

### Shared neural correlates of mental health and physical activity

#### Brain associations with mental health and physical activity

In simple CCA models (i.e., without accounting for the potentially shared variance in brain measures between physical activity and mental health), 2 and 3 significant canonical variate pairs were observed, respectively, for brain-mental health (*r*_1_ = 0.16, *p*_1_ = 0.001; *r*_2_ = 0.12, *p*_2_ = 0.001) and brain-physical activity associations (*r*_1_ = 0.23, *p*_1_ = 0.001; *r*_2_ = 0.15, *p*_2_ = 0.001; *r*_3_ = 0.13, *p*_3_ = 0.047). When controlling for the shared variance in brain measures between mental health and physical activity (i.e., unique models), we found 2 significant canonical variates for both brain-mental health (*r*_1_ = 0.14, *p*_1_ = 0.001; *r*_2_ = 0.12, *p*_2_ = 0.002) and brain-physical activity associations (*r*_1_ = 0.21, *p*_1_ = 0.001; *r*_2_ = 0.15, *p*_2_ = 0.001), with slightly decreased canonical correlation coefficients comparing to those from the simple models. The canonical variates for brain measures after accounting for the shared variance (i.e., from the unique models) mapped well with those from the simple models, as indicated by correlation coefficients between brain canonical variables (i.e., *U*; all *r*’s >0.96; see full results in Table S2). These results are in line with our expectations that both mental health and physical activity are closely associated with the functional networks under investigation and that mental health and physical activity have shared variance in the functioning of these networks, as indicated by the reduced canonical correlation coefficients from the unique models.

#### Canonical loadings of brain measures

**Fig. 2:**
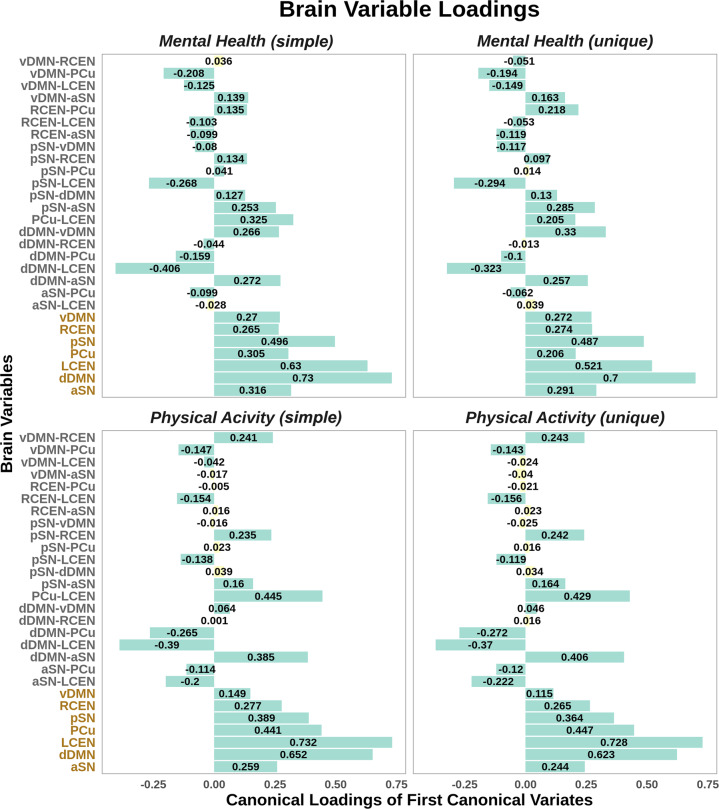
Canonical loadings of brain measures on the first canonical variate pair. These loadings represent the linear correlation between the original brain measures (Y) and the first canonical variate pair (U) per model. Color coding was made for brain variable names along the *Y* axis (i.e., subnetwork edges in gray with “-” between subnetwork names and amplitude in orange), and for the bars representing canonical loadings (i.e., significance in cyan, insignificance in yellow). Simple and unique models differ in whether the model accounted for the shared variance in brain measures between mental health and physical activity. vDMN ventral default mode network, R/L CEN right/left central executive network, PCu precuneus, a/p SN anterior/posterior salience network.

Findings from the post-hoc analyses on the significant canonical variates showed considerable overlap between the brain measures with significant loadings associated with mental health and with physical activity. Overall, the amplitude of subnetworks that indicates the overall signal fluctuations in each subnetwork over time loaded higher than the edges, with the highest loadings on the amplitude of dorsal DMN or left CEN for the first canonical variates in both the mental health and physical activity models (permuted *p* values ≤ 0.001; Fig. 2). This suggested that the magnitude of fluctuations of intra-network signal (i.e., amplitude) had higher contributions to the observed canonical associations with both mental health and physical activity than the inter-network connectivity strength. The similar loading pattern of all brain measures was largely retained even when the shared variance was partially out for mental health and for physical activity, respectively, in the unique models (permuted *p* values ≤ 0.035). In addition to amplitude, connectivity between the dorsal DMN and left CEN (i.e., subnetwork edge) also exhibited statistically significant loadings in the first canonical variates of both the mental health and the physical activity models (simple and unique models; permuted *p* values ≤ 0.001; Fig. 2). The brain variable loadings for the second canonical variate were highly similar between simple and unique models for either the mental health or physical activity models, with the amplitude and subnetwork edges showing evenly important involvement. Yet, the patterns of these loadings differed between the mental health and physical activity model (Fig. S2).

#### Canonical loadings of mental health questions and physical activities

Canonical loadings of both individual mental health questions and physical activity types for the first canonical variate pair also exhibited similar patterns between the simple and unique models (Fig. S3). In the models for brain-mental health associations, “risk taking” and “ever irritable/ argumentative for 2 days” had the highest loadings in both simple and unique models, whereas in the models for brain-physical activity associations, “walking” in all time windows (i.e., overall, weekdays, weekend) showed the greatest importance across models. Interestingly, among all physical activity types, only “sleep” showed the opposite direction in canonical loadings (Fig. S3).

### Genetic correlations

#### Genetic correlations for individual phenotypes

**Fig. 3:**
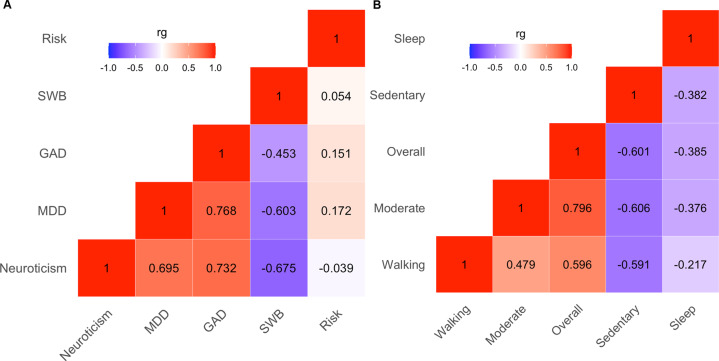
Heatmap of bivariate genetic correlations. Pairwise genetic correlations were calculated separately for phenotypes of mental health (**A**) and those of physical activity (**B**). rg genetic correlation coefficient, GAD generalized anxiety disorder, MDD major depressive disorder, Risk risk tolerance, SWB subjective well-being.

Pairwise LDSR was performed separately for psychopathological phenotypes and physical activity phenotypes. All results were significant after controlling for multiple comparisons, except for the genetic correlations between neuroticism and risk-taking (*rg* = 0.039, *p* = 0.075), and between subjective well-being and risk-taking (*rg* = 0.054, *p* = 0.146; Fig. 3). Specifically, among psychopathological phenotypes, the smallest significant genetic correlation was observed for generalized anxiety disorder and risk-taking (*rg* = 0.151, *p* = 2.69e-05, FDR-corrected *p* value = 3.36e-05), and the largest effect was observed for MDD and generalized anxiety disorder (*rg* = 0.768, *p* = 2.62e-90, FDR-corrected *p* value = 1.31e-89). For physical activity, genetic correlations ranged from −0.217 (*p* = 1.03e-04, FDR-corrected *p* value = 1.03e-04) for sleep duration and walking, to 0.796 (*p* = 1.77e-31, FDR-corrected *p* value = 8.85e-31) for moderate and overall activity (Fig. 3). Physical activity phenotypes adjusted for sex and BMI largely recapitulated these results, with genetic correlations ranging from −0.209 (*p* = 2.49e-04, FDR-corrected *p* value = 2.49e-04) for sleep duration and walking, to 0.780 (*p* = 2.12e-23, FDR-corrected *p* value = 1.06e-22) for moderate and overall activities.

#### Genetic correlations for latent factors

**Fig. 4:**
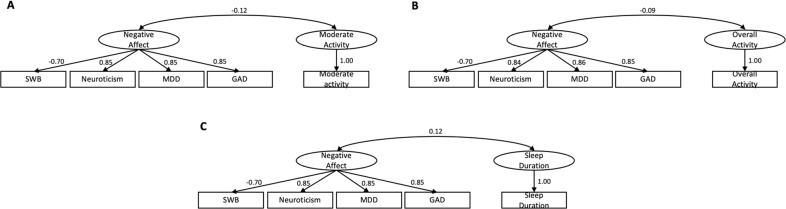
Genetic correlations between negative affect and physical activity phenotypes. Significant genetic correlations with negative affect were observed for moderate (**A**), overall activities (**B**), and sleep duration (**C**). The latent negative affect factor loaded positively on neuroticism, major depression disorder (MDD), and general anxiety disorder (GAD) phenotypes, but negatively on the subjective well-being (SWB) phenotype.

Analyses using gSEM across all mental health phenotypes returned results indicating suboptimal estimations with a low factor loading on risk-taking from the model for a general psychopathology latent factor (standardized loading = 0.065), which likely reflected its conceptually distinct construct from all other phenotypes (i.e., depression, anxiety, neuroticism, and subjective well-being). Additionally, initial gSEM results for physical activity phenotypes indicated overall poor model fit (CFI = 0.569, SRMR = 0.160; Fig. S4). Thus, we performed gSEM for psychopathological phenotypes grouped as one “negative affect” latent factor after excluding “risk-taking”, with an effective sample size of 571,170 and good model fit (CFI = 0.987, SRMR = 0.0535; Fig. S5). After correction for multiple testing, genetic variance in negative affect was significantly and positively correlated with sleep duration (*rg* = 0.121, *p* = 1.25e-05, FDR-corrected *p* = 1.25e-04), and negatively correlated with moderate (*rg* = −0.117, *p* = 1.33e-03, FDR-corrected *p* = 4.43e-03) and overall activity (*rg* = −0.085, *p* = 7.70e-04, FDR-corrected *p* = 3.85e-03; Fig. 4*;* also see the results for a latent negative affect factor including “risk-taking” in the Supplementary Results). No significant genetic correlation was observed between negative affect and sedentary activity or walking after multiple comparison corrections (|rgs| ≤ 0.062, *p* values ≥0.036, FDR-corrected *p* values ≥ 0.060; Table 3). Post-hoc analyses revealed that after adjustment for sex and BMI, only the genetic correlation between negative affect and sleep duration remained significant (*rg* = 0.122, *p* = 9.38e-06). Using LDSR, risk-taking was genetically correlated with both overall activity and walking (|rgs| ≥ 0.074, *p*s ≤ 0.020, *ps*FDR ≤ 0.040) but not moderate or sedentary activity or sleep duration (|rgs| ≤ 0.081*, p*s ≥ 0.092).

**Table 3:** Genetic correlations between mental health and physical activity phenotypes.

Mental health phenotype	Physical activity phenotype	*rg*	*p*	*pFDR*
Risk tolerance	Sleep duration	−0.029	0.433	0.447
	Sedentary	−0.028	0.447	0.447
	Moderate	0.081	0.092	0.131
	Walking	**0.115**	4.95e-3	0.012
	Overall	**0.074**	0.020	0.040
Negative affect	Sleep duration	**0.121**	1.25e-5	1.25e-4
	Sedentary	*−0.062*	0.036	0.060
	Moderate	**−0.117**	1.33e-3	4.43e-3
	Walking	−0.029	0.383	0.447
	Overall	**−0.085**	7.70e-4	3.85e-3

Significant results after FDR corrections were highlighted in bold.

*rg* genetic correlation, *pFDR* FDR-corrected *p* value.

#### Latent causal variable analysis

Results from the LCV analyses did not indicate causal relationships between any physical activity phenotypes and negative affect, in either direction (|GCPs| <0.425; *p*s > 0.163).

## Discussion

In this study, we investigated whether mental health and physical activity have shared brain and genetic architectures using the UK Biobank cohort. Our findings showed significant associations of mental health and physical activity separately with a set of brain measures that represent the connectivity strength and amplitude of subnetworks from the DMN, SN, and CEN. Critically, these significant associations exhibited highly similar patterns of brain variable loadings across mental health and physical activity models, even when the shared variance between these two constructs was accounted for, suggesting a potential overlap in brain network architecture between these two constructs. Further analyses examining genetic correlations for mental health and physical activity showed that negative affect exhibited significant genetic correlations with several physical activity types, of which sleep duration demonstrated the strongest genetic correlation that remained significant after controlling for BMI and sex effects. Together, these results support the presence of shared multivariate brain and genetic architectures between mental health and physical activity.

The three intrinsic brain networks, namely the DMN, SN, and CEN, have been consistently implicated in a wide range of psychiatric disorders including major depression and anxiety [10]. Interestingly, connections between or the configurations of these networks have also been associated with physical exercises [71, 72]. The current study therefore focused specifically on the subnetworks from these large-scale networks and used the amplitude and connectivity strength (i.e., subnetwork edge) to examine the associations of these networks with mental health and with physical activity, respectively. In line with the literature, we observed significant multivariate associations for all three networks with either mental health or physical activity, and significant loadings on most of the subnetworks. In particular, the dorsal DMN and left CEN showed the greatest involvement in the observed brain associations with both mental health and physical activity (Fig. 2). In this study, the dorsal DMN subnetwork primarily consisted of the PCC and the ventromedial prefrontal cortex (vmPFC), the two brain areas that are commonly considered as the core subsystem of the DMN [73]. Similarly, the subnetwork of the CEN here included the two most typical hub regions: the dorsolateral prefrontal cortex (dlPFC) and posterior parietal cortex for each individual hemisphere (Fig. S1). These major hubs of the DMN and CEN have been implicated in various mental illnesses including depression and anxiety. For example, the PCC and mPFC have been suggested to collectively support multiple cognitive functions including decision-making and memory [74], the impairment of which has often been reported in patients with major depression and anxiety disorders [75–77]. Additionally, the dlPFC is known to be involved in emotion regulation [78–80] and dysfunction of this region is often seen in abnormal processing of emotional experiences in patients with depressive and anxiety symptoms [81, 82]. As for physical activity, increased dlPFC activity has been observed after acute physical exercises in participants with higher scores in the Stroop test [83], whereas the involvement of the DMN subsystems in the medial temporal lobe including the hippocampus and its connection with the medial PFC is often observed in relation to enhanced memory after physical exercises [20, 84, 85]. In our findings, the amplitude of dorsal DMN and left CEN that indicated the magnitude of fluctuations of intra-network signal (i.e., variance in the connections between PCC and vmPFC or between dlPFC and PCC), as well as the connectivity strength between these two subnetworks, showed significantly high loadings for the most critical association between brain measures and mental health measures (i.e., the first canonical variate). These observations are in line with separate literature on mental health and physical activity, and provide empirical evidence that mental health and physical activity may share brain architecture involved in major cognitive functions.

Interestingly, mental health and physical activity also appear to have partially overlapping genetic architectures. In line with previous reports that internalizing problems are negatively genetically correlated with physical movement [45], we showed that a latent negative affect factor capturing genetic covariance between subjective well-being, neuroticism, major depressive disorder, and generalized anxiety disorder was negatively genetically correlated with overall physical activity as well as a more fine-grained phenotype of moderate activity, and positively genetically correlated with sleep duration. Protective effects of physical activity on mental health have long been documented, as have negative health consequences of psychiatric disorders [5–7, 86]. Here, we demonstrate that these relationships can be partially explained by shared genetic predisposition, although results from our latent causal variable analysis indicate that these associations do not reflect causal influences. Interestingly, with the adjustment of sex and BMI, only the correlation with sleep duration remained significant. Sleep duration also showed the lowest loading onto a latent physical activity genetic factor (Fig. S4). These results suggest that the sleep phenotype is somewhat distinct from the remaining physical activity phenotypes and that the shared genetic architecture between negative affect and sleep duration is more pronounced than that between negative affect and the degree of daily physical or sedentary activity. This is in line with the frequent documentation of symptomatic sleep disturbances across forms of psychopathology, including depression and anxiety [87, 88], even in children [89]. This also aligns well with the literature showing reduced sleep duration in older adults with depression and anxiety disorders [90–92].

Despite being the first to jointly investigate the shared brain network architecture and genetic basis of mental health and physical activity in a large population cohort, our study has some limitations. First, the brain measures in our study are derived from resting-state fMRI measures. Although our choice reflected a rich literature that has implicated these measures in both mental health and physical activity, addressing smaller-scale brain structures (e.g., specific regions) with relevant hypotheses and inclusion of multimodal brain measures such as structural gray matter volume, cortical thickness, and white matter integrity can provide complementary insights into brain architecture in relation to mental health and physical activity, and thus may be of interest for future investigations. Second, although the accelerometer recording took place prior to the acquisition of resting-state fMRI and mental health assessment for most of the participants in our study (i.e., 96%), the degrees of the time difference between these measurements varied greatly at the individual level (i.e., ranging between −473 and 2281 days). This time discrepancy was accounted for in all CCA models as a covariate and caution should be taken when interpreting the observed brain associations with reference to time effects. It should also be noted that the mean age of the current sample is relatively high as the UKB cohort comprises predominantly middle-to-late aged individuals. Our findings therefore should be interpreted carefully in the relevant context. Lastly, socioeconomic variables including education attainment can be relevant to mental health and physical activity phenotypes, and the inclusion of these variables as confounding factors may be considered in future investigations.

In conclusion, our study jointly analyzed resting-state network measures and genetic correlations in a large cohort to test the hypothesis of a shared neurobiological basis for mental health and physical activity. Our findings revealed that multivariate patterns of brain correlates were highly similar between mental health and physical activity and highlighted genetic correlations between mental health (negative affect) and overall physical activity, moderate activity levels, and sleep duration. Taken together, these findings point towards neural and genetic mechanisms that may subserve the protective influence of physical exercise and sleep on mental health.

## Supplementary information

SUPPLEMENTAL MATERIAL

## Data Availability

The UK Biobank data used in this study can be accessed by researchers upon application (https://www.ukbiobank.ac.uk/register-apply). We also shared the derived data from statistical analyses and code for producing the figures on the Open Science Framework (https://osf.io/p3fzv/). To note, the GWAS summary statistics used in the current study are hosted elsewhere, for which we only shared the downloading links.

## Code availability

A machine learning algorithm shared on github was used to extract physical activity measures (https://github.com/activityMonitoring/biobankAccelerometerAnalysis). LCV was performed using the open-source LCV software (https://github.com/lukejoconnor/LCV). The Matlab code for running permutation inference for CCA are also available on github, including the new extension for testing canonical variable loadings (https://github.com/andersonwinkler/PermCCA). We performed Pearson’s correlations and imputations in R (version 4.1.0) [70], using basic *stat* function and *MICE* package [47], respectively. Genetic analyses including LDSR and gSEM were performed using the GenomicSEM package also in R [66], with code available on github (https://github.com/GenomicSEM/GenomicSEM). All the code used for statistical analyses and visualizations in this study is shared on the Open Science Framework (https://osf.io/p3fzv/).
