# Supplementary material for: Shared brain and genetic architectures between mental health and physical activity
Source: Transl Psychiatry. 2022 Oct 3;12:428. doi: 10.1038/s41398-022-02172-w (PMC9530213; doi:10.1038/s41398-022-02172-w)
Supplement: Supplementary file 1 — SUPPLEMENTAL MATERIAL [file 41398_2022_2172_MOESM1_ESM.docx]

**Supplementary Results**

**Identification of functional networks**

To identify the best matching components corresponding to the seven functional parcels from the Stanford FIND atlas, we considered two different dimensionalities of independent component analysis (ICA): ICA25 with 25 components and ICA100 with 100 components. In contrast to ICA25, components from ICA100 exhibited higher spatial correlations with the FIND atlas on average (mean r = 0.37) with less spatial overlap between the components (*Figure S1*). Thus, seven ICA components from ICA100 were identified as the network nodes in our study sample.

**Genetic analyses**

*gSEM for mental health phenotypes including “risk taking”.*

Models in which “risk taking” loaded onto the negative affect factor produced similar results as have been observed in the model excluding this phenotype, with significant genetic correlations observed between negative affect and moderate, overall, and sedentary activity and sleep duration (|rgs| ≥ 0.0855, ps ≤ 1.60e-03), but not walking (rg = -0.028, p = 0.402). After FDR corrections, only moderate, overall, and sedentary activity, as well as sleep duration, remained statistically significant (FDR-corrected p-values ≤ 0.0458).

*Major Depressive Disorder GWAS Meta-Analysis.*

In an effort to recapitulate the most recent GWAS meta-analysis of Major Depressive Disorder (MDD; Levey et al., 2021), we meta-analyzed the case-control summary statistics from the UK Biobank and Psychiatric Genetics Consortium (N=500,199; Howard et al., 2019) and those from the Million Veteran Program (N=250,215; Levey et al., 2021) using the METAL software. This analysis resulted in an effective sample size of 145,018, and 100 significant independent loci were identified.

**Figures and Tables**


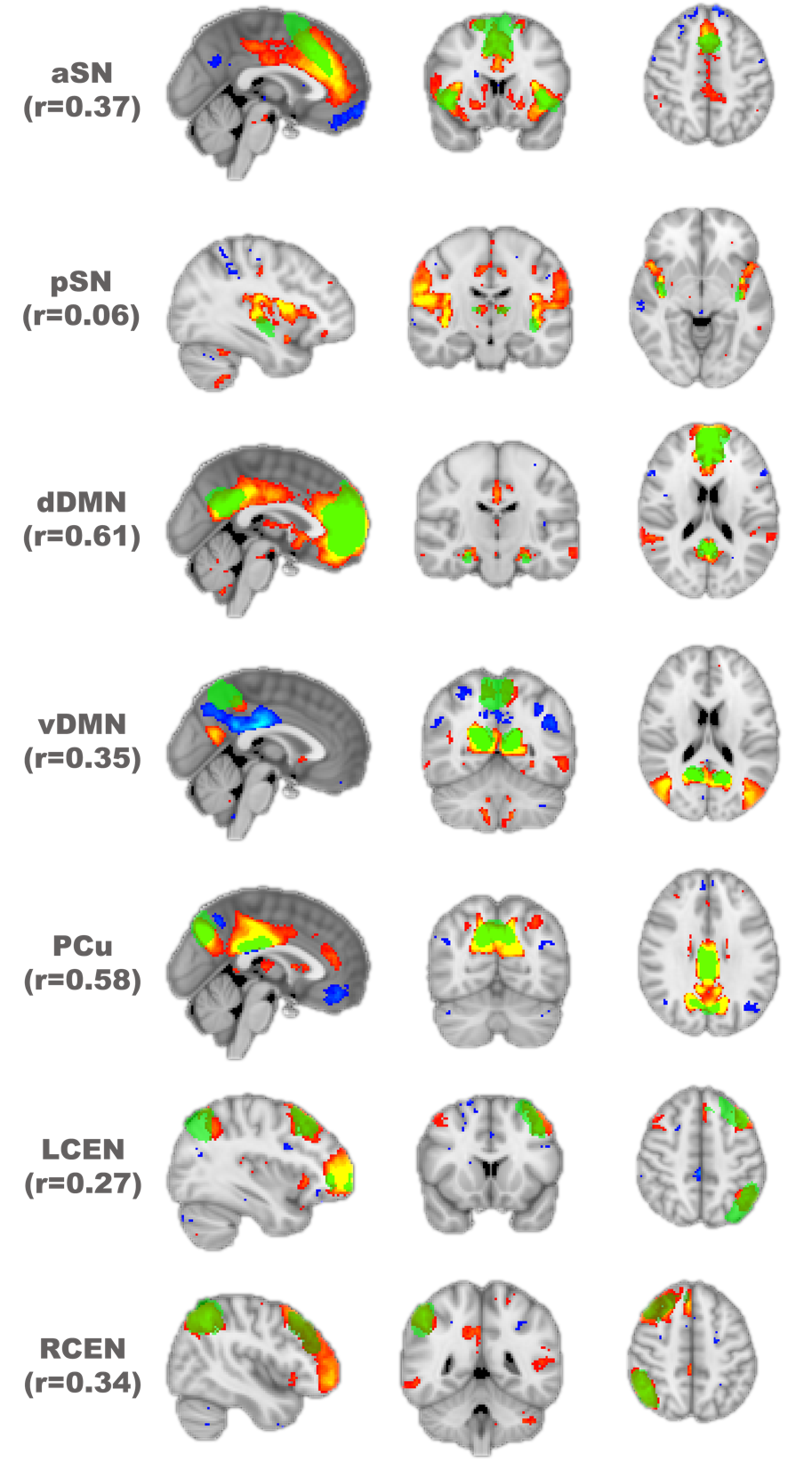


**Figure S1. Preselected brain network nodes.** Seven components from a group-ICA (dimensionality = 100) were selected based on their spatial correlations with the default mode, salience, and central executive networks (DMN, SN, CEN) from the Stanford FIND atlas. These networks from the atlas (in green) are imposed on the selected ICA nodes, with a threshold of Z>3 for illustration purposes. a/p SN = anterior/posterior salience network, d/v DMN = dorsal/ventral default mode network, PCu = precuneus, L/R CEN = left/right central executive network.


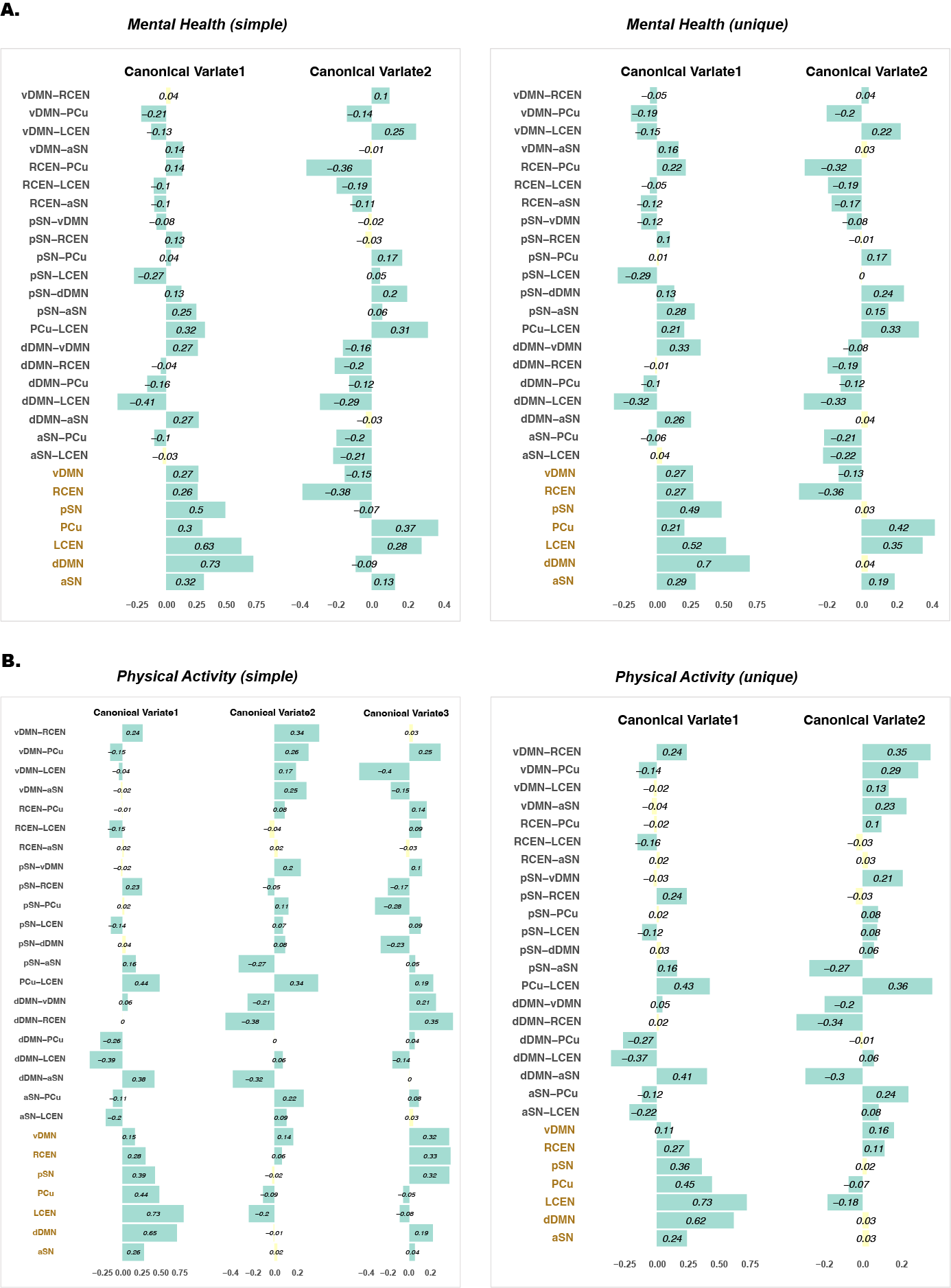


**Figure S2. Canonical loadings of brain measures on all significant canonical variates.** Canonical loadings of brain measures on all significant canonical variates. These loadings represent the linear correlation between the original brain measures and all significant canonical variates per model. Unique and simple models for brain associations with mental health (A) and physical activity (B) are illustrated. Simple and unique models differ in whether the model accounted for the shared variance in brain measures between mental health and physical activity. Color coding was made for brain variable names along the Y axis (i.e., node edges in gray with “-” between node names and amplitude in orange), and for the bars representing canonical loadings (i.e., significance in cyan, insignificance in yellow). vDMN = ventral default mode network; R/L CEN = right/left central executive network; PCu = precuneus; a/p SN = anterior/posterior salience network.


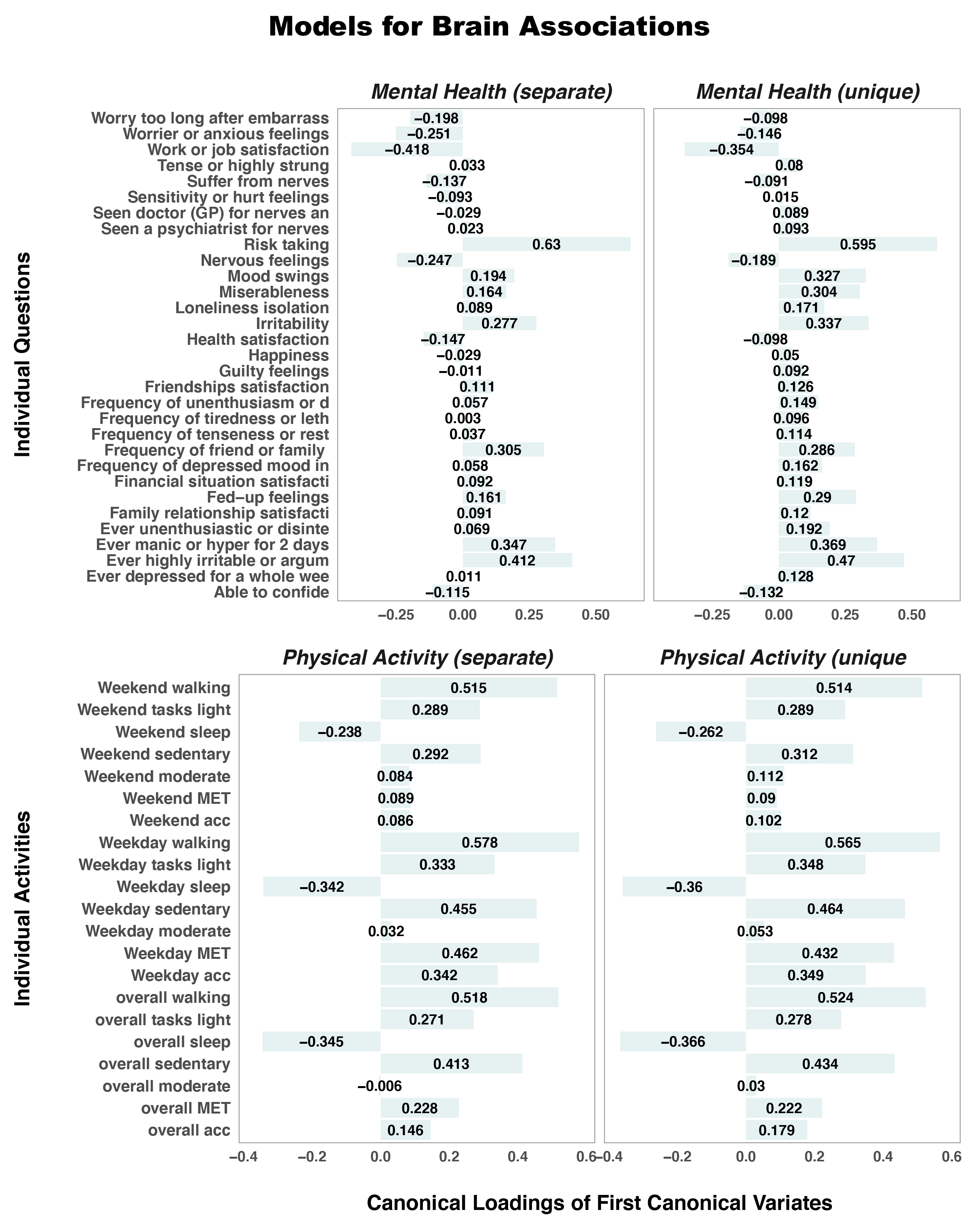


**Figure S3. Canonical loadings of mental health questions and physical activity types.** Canonical loadings are shown for the first canonical variates from all models. These loadings represent the linear correlation between the original mental health or physical activity measures (*Y*) and all significant canonical variate ($U$) per model. Simple and unique models differ in whether the model accounted for the shared variance in brain measures between mental health and physical activity. Please note, descriptions of each individual mental health question are shortened for illustration purposes. Complete descriptions can be found in *Table S1*.

**
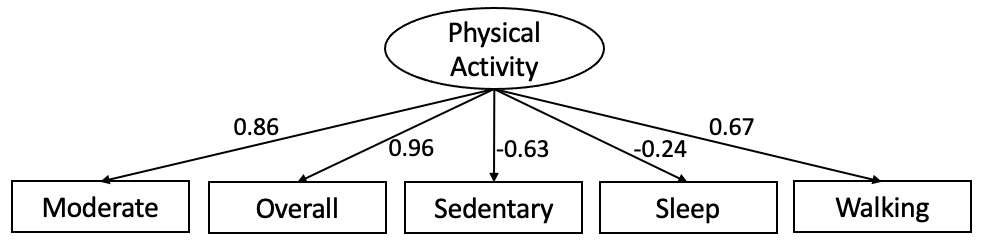
**

**Figure S4. Single genetic factor model of physical activity phenotypes.** Note, estimates represent standardized factor loadings. CFI = 0.569, SRMR = 0.160.


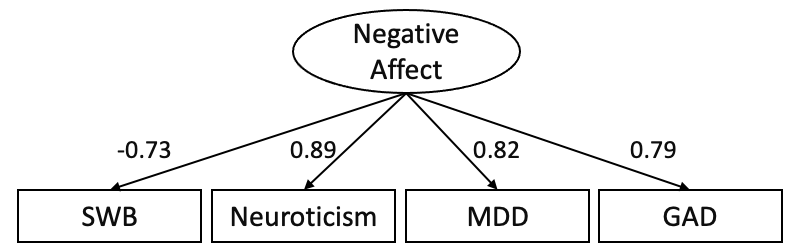


**Figure S5. Single genetic factor model of negative affect phenotypes (excluding “risk factor”).** Note*,* estimates represent standardized factor loadings. CFI = 0.987, SRMR = 0.0535. SWB = subjective well-being. MDD = major depressive disorder. GAD = generalized anxiety disorder.

**Table S1.** Principal component loadings of mental health questionnaire items.

| **Mental Health Questions (variable ID)** | **Principal Components (explained variance%)** | | | | | | |
| --- | --- | --- | --- | --- | --- | --- | --- |
|  | **PC1 (22.47)** | **PC2 (6.77)** | **PC3 (5.72)** | **PC4 (4.66)** | **PC5 (4.08)** | **PC6 (3.98)** | **PC7 (3.39)** |
| Frequency of friend or family visits (1031) | -0.047 | 0.164 | -0.072 | 0.127 | -0.355 | 0.066 | -0.378 |
| Mood swings (1920) | -0.253 | -0.072 | -0.018 | -0.225 | -0.081 | 0.241 | 0.002 |
| Miserableness (1930) | -0.245 | -0.059 | 0.022 | -0.196 | -0.036 | 0.289 | -0.029 |
| Irritability (1940) | -0.174 | -0.063 | -0.114 | -0.087 | -0.291 | 0.042 | 0.103 |
| Sensitivity / hurt feelings (1950) | -0.185 | -0.227 | -0.176 | 0.085 | -0.032 | 0.252 | 0.166 |
| Fed-up feelings (1960) | -0.248 | 0.023 | -0.042 | -0.209 | -0.028 | 0.248 | -0.072 |
| Nervous feelings (1970) | -0.178 | -0.272 | -0.239 | 0.169 | -0.022 | -0.301 | -0.079 |
| Worrier / anxious feelings (1980) | -0.194 | -0.271 | -0.199 | 0.113 | 0.084 | 0.069 | 0.11 |
| Tense / ‘highly strung’ (1990) | -0.186 | -0.182 | -0.135 | 0.01 | -0.18 | -0.367 | -0.093 |
| Worry too long after embarrassment (2000) | -0.184 | -0.214 | -0.217 | 0.145 | 0.038 | 0.204 | 0.11 |
| Suffer from ‘nerves’ (2010) | -0.185 | -0.189 | -0.155 | 0.108 | -0.058 | -0.403 | -0.101 |
| Loneliness, isolation (2020) | -0.197 | 0.127 | -0.022 | -0.061 | 0.001 | 0.083 | -0.215 |
| Guilty feelings (2030) | -0.194 | -0.124 | -0.109 | 0.007 | -0.024 | 0.173 | 0.101 |
| Risk taking (2040) | 0.004 | 0.123 | 0.161 | -0.177 | -0.466 | -0.046 | 0.202 |
| Frequency of depressed mood in last 2 weeks (2050) | -0.247 | 0.139 | 0.043 | -0.282 | 0.157 | -0.116 | -0.161 |
| Frequency of unenthusiasm / disinterest in last 2 weeks (2060) | -0.223 | 0.176 | 0.074 | -0.291 | 0.178 | -0.151 | -0.182 |
| Frequency of tenseness / restlessness in last 2 weeks (2070) | -0.223 | 0.035 | -0.011 | -0.227 | 0.073 | -0.273 | -0.103 |
| Frequency of tiredness / lethargy in last 2 weeks (2080) | -0.208 | 0.094 | 0.047 | -0.242 | 0.196 | -0.098 | 0.12 |
| Seen doctor (GP) for nerves, anxiety, tension, or depression (2090) | -0.188 | -0.144 | 0.367 | 0.208 | 0.069 | -0.008 | -0.118 |
| Seen a psychiatrist for nerves, anxiety, tension, or depression (2100) | -0.138 | -0.091 | 0.295 | 0.217 | -0.02 | -0.178 | -0.063 |
| Able to confide (2110) | 0.094 | -0.241 | 0.12 | -0.142 | 0.123 | -0.093 | 0.284 |
| Happiness (4526) | -0.227 | 0.272 | -0.093 | 0.151 | 0.109 | 0.059 | 0.03 |
| Work / job satisfaction (4537) | -0.019 | 0.01 | -0.082 | 0.075 | 0.398 | 0.045 | 0.054 |
| Health satisfaction (4548) | -0.153 | 0.219 | -0.024 | 0.086 | 0.25 | -0.149 | 0.401 |
| Family relationship satisfaction (4559) | -0.158 | 0.353 | -0.085 | 0.291 | -0.06 | 0.001 | 0.03 |
| Friendships satisfaction (4570) | -0.147 | 0.34 | -0.144 | 0.319 | -0.124 | 0.033 | -0.014 |
| Financial situation satisfaction (4581) | -0.136 | 0.259 | 0.028 | 0.143 | 0.022 | -0.066 | 0.363 |
| Ever depressed for a whole week (4598) | -0.158 | -0.119 | 0.43 | 0.242 | 0.064 | 0.146 | -0.086 |
| Ever unenthusiastic / disinterested for a whole week (4631) | -0.19 | -0.094 | 0.432 | 0.17 | 0.045 | 0.083 | -0.078 |
| Ever manic / hyper for 2 days (4642) | -0.09 | 0.002 | 0.183 | -0.025 | -0.249 | -0.177 | 0.317 |
| Ever highly irritable / argumentative for 2 days (4653) | -0.146 | -0.021 | 0.169 | -0.11 | -0.276 | -0.028 | 0.27 |

Principal component loadings of the first seven components (i.e., PC1-PC7), the scores of which for each participant were used as indications for mental health measures. These components altogether explained 51.05% variance in the data. Four-digit numbers reported after each individual question in parentheses are variable IDs in the UKB Data Showcase. Cells with negative values are highlighted with light gray shading.

**Table S2.** Pairwise correlations of brain variable scores.

|  | **Simple Models** | | | | | |
| --- | --- | --- | --- | --- | --- | --- |
|  | Mental Health | | | Physical Activity | | |
|  |  | CV1 | CV2 | CV1 | CV2 | CV3 |
| **Unique**  **Models** | CV1 | **0.980** | 0.483 | **0.998** | -0.265 | 0.131 |
|  | CV2 | 0.138 | **0.969** | -0.192 | **0.994** | 0.029 |
|  | CV3 | - | - | 0.202 | -0.201 | **0.995** |

Each canonical variate for brain measures (*U*) was correlated between simple (i.e., not accounting for shared variance) and unique models, for all significant canonical variates (i.e., two in mental health models, CV1 and CV2; three in physical activity models, CV1 to CV3). Highest correlation coefficients are shown in bold.

**References**

1. Levey DF, Stein MB, Wendt FR, Pathak GA, Zhou H, Aslan M, *et al.* (2021): Bi-ancestral depression GWAS in the Million Veteran Program and meta-analysis in >1.2 million individuals highlight new therapeutic directions. *Nat Neurosci*. https://doi.org/10.1038/s41593-021-00860-2

2. Howard DM, Adams MJ, Clarke TK, Hafferty JD, Gibson J, Shirali M, *et al.* (2019): Genome-wide meta-analysis of depression identifies 102 independent variants and highlights the importance of the prefrontal brain regions. *Nat Neurosci*. https://doi.org/10.1038/s41593-018-0326-7
